# CarboGrove: a resource of glycan-binding specificities through analyzed glycan-array datasets from all platforms

**DOI:** 10.1093/glycob/cwac022

**Published:** 2022-03-29

**Authors:** Zachary L Klamer, Chelsea M Harris, Jonathan M Beirne, Jessica E Kelly, Jian Zhang, Brian B Haab

**Affiliations:** Department of Cancer and Cell Biology, Van Andel Institute, 333 Bostwick Ave NE, Grand Rapids, MI 49503, United States; Z Biotech, Aurora, CO, United States; Z Biotech, Aurora, CO, United States; Z Biotech, Aurora, CO, United States; Z Biotech, Aurora, CO, United States; Department of Cancer and Cell Biology, Van Andel Institute, 333 Bostwick Ave NE, Grand Rapids, MI 49503, United States

**Keywords:** binding specificity, database, glycan-binding protein, lectin, microarray

## Abstract

Glycan arrays continue to be the primary resource for determining the glycan-binding specificity of proteins. The volume and diversity of glycan-array data are increasing, but no common method and resource exist to analyze, integrate, and use the available data. To meet this need, we developed a resource of analyzed glycan-array data called CarboGrove. Using the ability to process and interpret data from any type of glycan array, we populated the database with the results from 35 types of glycan arrays, 13 glycan families, 5 experimental methods, and 19 laboratories or companies. In meta-analyses of glycan-binding proteins, we observed glycan-binding specificities that were not uncovered from single sources. In addition, we confirmed the ability to efficiently optimize selections of glycan-binding proteins to be used in experiments for discriminating between closely related motifs. Through descriptive reports and a programmatically accessible Application Programming Interface, CarboGrove yields unprecedented access to the wealth of glycan-array data being produced and powerful capabilities for both experimentalists and bioinformaticians.

## Introduction

Glycan arrays are being produced and used by more labs than ever before. After the first reports of glycan arrays in 2004 (Blixt et al. 2004; Bryan et al. 2004), just a handful of laboratories worked on the technology for about the next decade. The main provider of the technology was the Consortium for Functional Glycomics (CFG). The CFG array, which used the planar array method that had been established for DNA and protein arrays, contained glycans that represented a broad survey of the known, important motifs in mammalian biology. The significance of the CFG resource was that it provided access to researchers who could not produce arrays themselves, primarily by reason of the cost and difficulty of synthesizing glycans. It was the large number of experiments performed on this platform that established the value of glycan-array technology and stimulated further developments in the field, including in experimental methods, bioinformatics tools, and methods of glycan synthesis. The CFG data were the sole data source for multiple bioinformatics efforts in the analysis of glycan array data (Porter et al. 2010; Cholleti et al. 2012; Cao et al. 2019; Coff et al. 2020).

But no single array could meet the needs of every study. The diversity and number of structures present among various classes of glycans and organism types are too great even for the largest array. This situation drove researchers to develop arrays with content organized around specific fields of research. For example, plant biologists and microbial biologists each developed glycan arrays relevant to their fields (Ruprecht et al. 2017; Geissner et al. 2019), and researchers studying sialic acids developed arrays with a wide range of variants of that feature (Song et al. 2011). Other features of specialized content include glycosaminoglycans (Horton et al. 2020; Chopra et al. 2021), glycopeptides (Hinou et al. 2019; Mende et al. 2020; Nason et al. 2021), and human milk oligosaccharides (Prudden et al. 2017). Improved synthetic strategies and automated synthesizers helped address the major hurdle of glycan production (Zhang et al. 2018; Li, Liu, et al. 2019b), in combination with focused production around specific types of glycans. Furthermore, researchers developed experimental alternatives to the planar array. The novel technologies—methods involving mass spectrometry (Kitov et al. 2019) or bacteriophage display (Sojitra et al. 2021), for example—provide complementary information and capabilities to the planar array and allow dispersion of the methods to a greater number of laboratories.

All of these developments have resulted in an expanding variety of glycan-array data available for study. Bioinformatics methods that could capture and use all available glycan-array data, regardless of source and content, could serve many purposes, from learning more about the specificity of a particular protein, to finding lectins with a pre-defined specificity, to larger-scale, integrative studies in glycobiology. Such analyses cannot be done manually, given the complexity of glycans and protein–glycan interactions, as well as the complexity of integrating information over many data points from the array. The data from the various sources must be analyzed and interpreted with a common system.

Several resources currently provide glycan-array data in either raw or analyzed form: CFG, Lectin Frontier Database (LFDB; Hirabayashi et al. 2015), Multiple Carbohydrate Alignment with Weights Database (MCAW-DB; Hosoda et al. 2018), and Glycan Microarray Database (GlyMDB; Cao et al. 2019). These resources represent valuable advances in the field, but they have limited value as a general resource for non-bioinformaticians. One limitation is that each provides data for only a single type of array, either CFG (CFG, MCAW-DB, and GlyMDB) or frontal-affinity chromatography (LFDB). Further, there are resources which provide powerful visualization tools, such as the GLAD (GLycan Array Dashboard) employed by GlyMDB, but limited or incomplete interpretation of the data. For example, MCAW-DB gives an alignment of top-binding glycans, which gives clues about features associated with binding, but it does not provide the context of their binding strength relative to others, which is necessary to achieve a complete picture. In general, the discernment of the specificity of a glycan-binding protein requires significant, additional analysis on the part of the researcher. The need for algorithms to discern the complex, fine specificities of glycan-binding proteins is clear, given the sensitivity of binding to minor differences in glycans such as the position of the epitope on *N*-glycan branches (Li, Guan, et al. 2019a; Wang et al. 2021).

Given software to reliably interpret data from any platform, a resource could be built that provides common access to glycan-array information across the many sources that are now available. We recently introduced the MotifFinder software (Klamer et al. 2017; Klamer and Haab 2021) to meet the analysis need. We demonstrated earlier that the algorithm delivers a detailed and accurate analysis of the specificity of a glycan-binding protein and that it can perform the analyses through the integration of data from distinct platforms. These previous developments suggested an approach to unify the analysis and usage of glycan array data. In the present work, we explored whether a database system driven by the MotifFinder engine could meet the need for a unified glycan-array resource.

## Results

### Achieving common data processing across platforms and array types

**Fig. 1:**
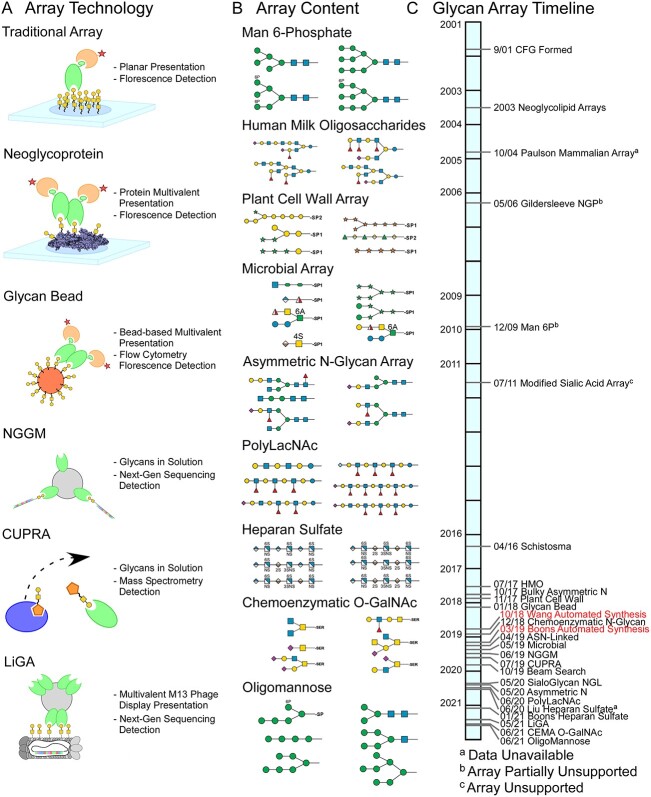
Diversity of glycan-array technology and content. A) Several technologies in addition to the planar array are now used to probe glycan arrays. The arrays differ in their modes of glycan or protein presentation and in methods of quantification. B) The sets of glycans contained in the arrays represent diverse types of structures and organisms. C) The rate of development of new arrays has increased since 2016, punctuated by significant advances in glycan-synthesis technology (red text). Monosaccharide symbols follow the SNFG (Symbol Nomenclature for Glycans) system (PMID 26543186, Glycobiology 25: 1323–1324, 2015) details at NCBI.

The available glycan-array data cover several approaches to detection and quantification (Fig. 1A). This diversity represents a challenge when collating data into a common platform, but it also provides complementary information from the strengths and limitations of each platform. The planar array uses robust methods that were established for DNA arrays and thus has been a workhorse for many labs, but it has the limitation that nonspecific or reduced binding can occur from the linker (Grant et al. 2014), the surface (Li et al. 2021), or the tagging of the glycan-binding protein (Kitova et al. 2019). Most embodiments do not account for glycan density or kinetics. The newer technologies provide solution-phase kinetics (Kitov et al. 2019; Sojitra et al. 2021), incorporation of density as a controllable parameter (Mende et al. 2020), or display on a cell-surface context (Büll et al. 2021), but they require specialized methods or equipment.

The available data also contain a great diversity of glycans (Fig. 1B). The CFG array was heavily weighted toward mammalian glycans, but technology developers have branched out into microbial, plant cell wall, and other nonmammalian glycans. In addition, improved synthesis technologies have resulted in glycans with increased complexity and a broader variety of monosaccharides, as well as a variety of glycosaminoglycans. These developments have resulted in an increased frequency in reports on new glycan arrays (Fig. 1C, Supplementary Table I).

We sought to develop a system that provides a common mode of analysis for all available data. To account for the diversity in glycans, we utilized MotifFinder’s glycan parser that translates text representations of all types of monosaccharide names and their connections in standard CFG-like notation to graph structures used by the program. To enable the processing of data from any type of array or platform, we developed the analysis algorithm to be independent of scale or range but require only a quantitative value corresponding to each glycan in the array positively associated with binding.

**Fig. 2:**
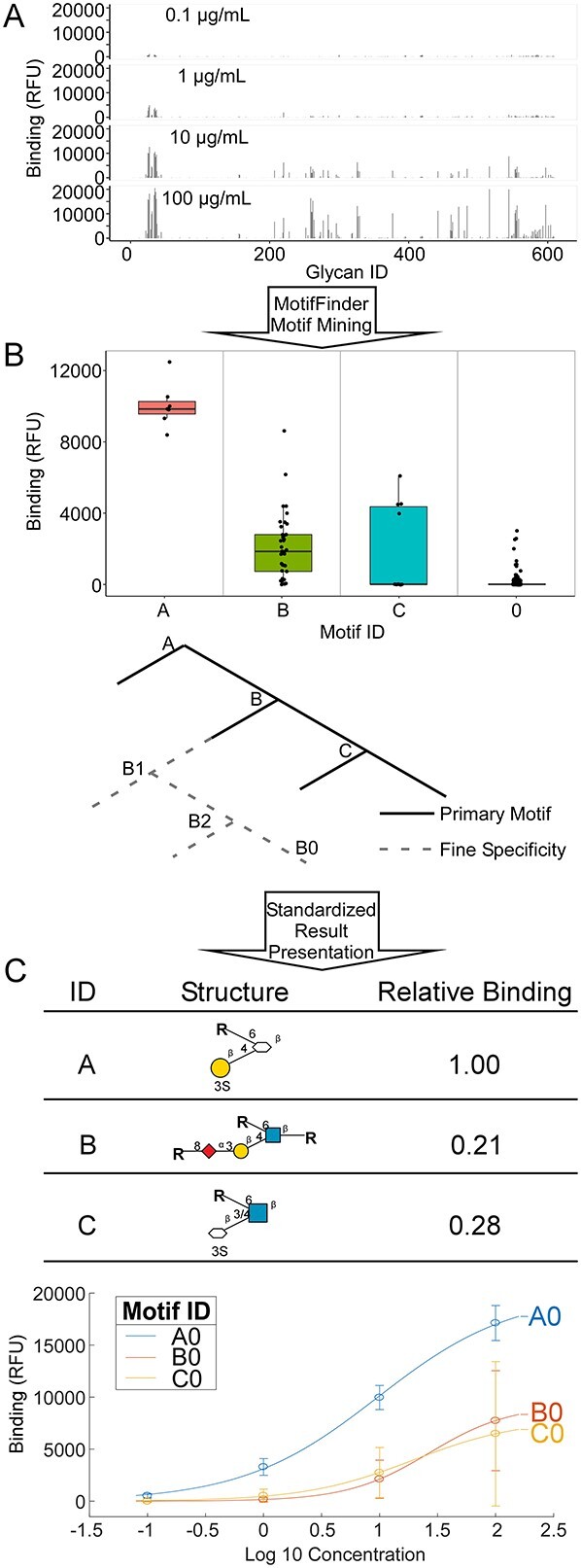
Standardized analysis and output. A) MotifFinder analyzes glycan-array data from multiple incubation concentrations, where available, of a given glycan-binding protein. B) The program identifies the family of motifs that represents the specificity of the protein, organized into primary motifs and the subtrees of fine-specificity motifs. C) Among the several visualizations in the output are tabular descriptions and binding curves for each motif. Monosaccharide symbols follow the SNFG (Symbol Nomenclature for Glycans) system (PMID 26543186, Glycobiology 25: 1323–1324, 2015) details at NCBI.

The various glycan-array data could then be processed in our MotifFinder algorithm for identifying the motifs—patterns within glycans—that best describe the specificity of the glycan-binding protein applied to the array. The algorithm uses data from multiple concentrations of the protein, if available, to give more accurate results than possible from one concentration (Klamer and Haab 2021) (Fig. 2A). The family of motifs that defines the specificity (referred to as the model) is arranged into 2 types: the primary motifs, which represent distinct structural categories, and the fine-specificity motifs, which represent gradations in binding within the primary motifs (Fig. 2B). The model is visualized in various ways to assist user interpretation (Fig. 2C). The consistent output across all datasets, regardless of platform or type of glycans, is a critical component of enabling cross-dataset comparisons and searches.

**Fig. 3:**
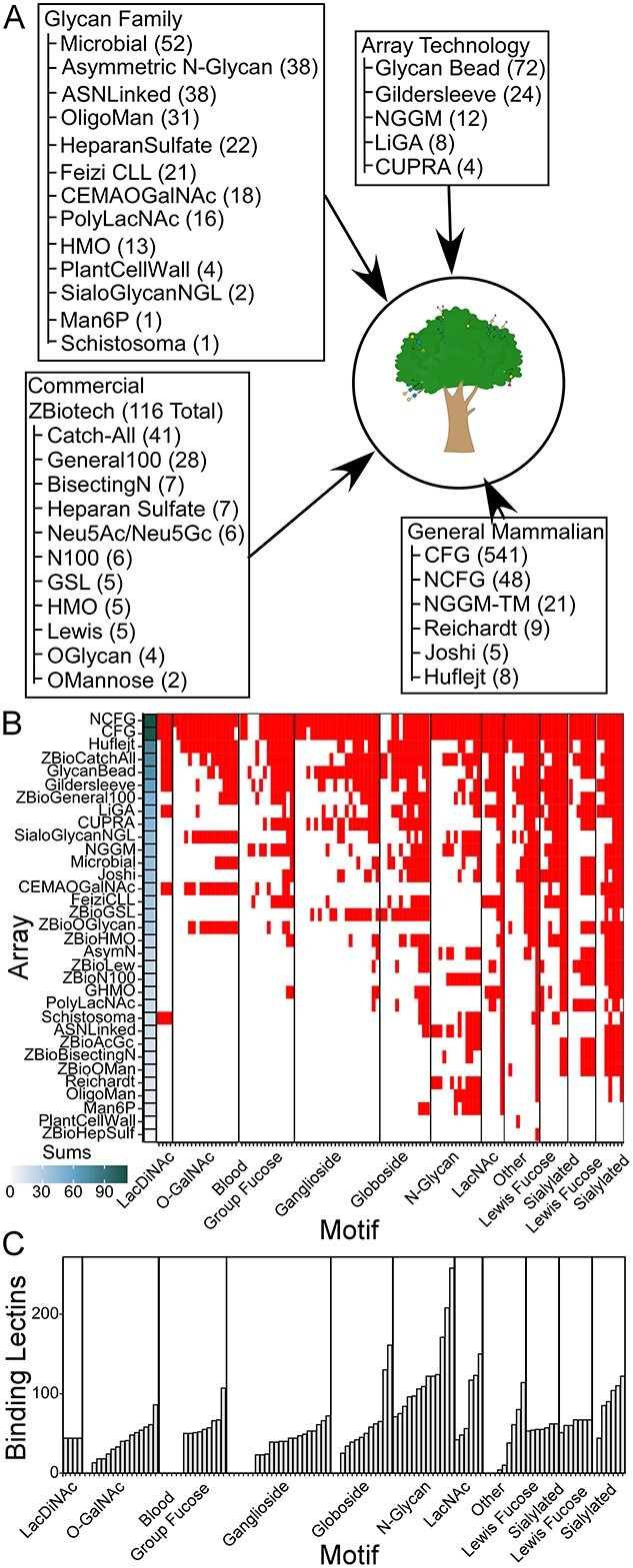
Breadth of representation of array types of glycan-binding specificities. A) The collection covers a wide variety of glycan families, array providers, and technologies. B) Motif coverage across the arrays. Nearly all motifs are represented on at least 1 array. C) Motif coverage of the glycan-binding proteins.

We tested the ability of the algorithm to process and organize glycan-array data from 35 different types of arrays, 13 different glycan families, 5 experimental methods, and 19 laboratories or companies (Fig. 3A, Supplementary Table II). These included publicly available data as well as unpublished data (Supplementary Tables II and III). The number of contributions from each provider ranged from 1 to 541 datasets, for a total of 1,125 datasets (Supplementary Table IV). MotifFinder was able to produce a model for each of the glycan-binding proteins with only minor adjustments in formatting required for some datasets.

We then assembled the models into a relational database called CarboGrove (Fig. 3A). This collection promises to cover a much broader range of glycans and the glycan-binding proteins than any single resource. To evaluate the scope of the database, we defined 118 different motifs from 11 families based on a set obtained from the GlyGen resource (York et al. 2019) with the addition of motifs covering the major core types (Supplementary Tables V and VI). This list is not exhaustive but provides an initial, unbiased survey of the breadth of the database. An analysis of the glycans on the arrays showed that all motifs were represented on at least one array and that some were on nearly every array (Fig. 3B). The arrays had a broader range of inclusion of motifs, ranging from only 1 to 112, reflecting the variation in purposes of the arrays.

To determine whether the glycan-binding proteins in the database cover a broad range of specificities, we used the model for each protein to predict binding to each of 1,803 glycans that spanned all arrays. From the resulting values, we assessed binding to each of the 118 motifs using a motif score (Klamer and Haab 2021). All but 19 of the 118 motifs are bound (motif score > 2) by 4 or more glycan-binding proteins (Fig. 3C, Supplementary Table V). The 19 motifs not bound by any proteins represent less-common features such as type 3 A antigen. Very common motifs such as *N*-glycan and biantennary *N*-glycan are bound by many glycan-binding proteins: 258 and 208, respectively. These differences reflect both the prevalence of the motif in biology and the amount of research centered on the motif.

### Accessing and analyzing glycan arrays across platforms

The collection of analyzed data potentially offers access to detailed, accurate information about the specificity of any given glycan-binding protein. We sought to enable such searches through a system of matching user-specified terms with all relevant datasets. This task involved accounting for variability in the conventions in common names, abbreviations, and the use of the terms lectin and agglutinin. To address this difficulty, we included multiple aliases for each protein and allowed relevant results to be returned even when a search does not match the primary name in the database.

**Fig. 4:**
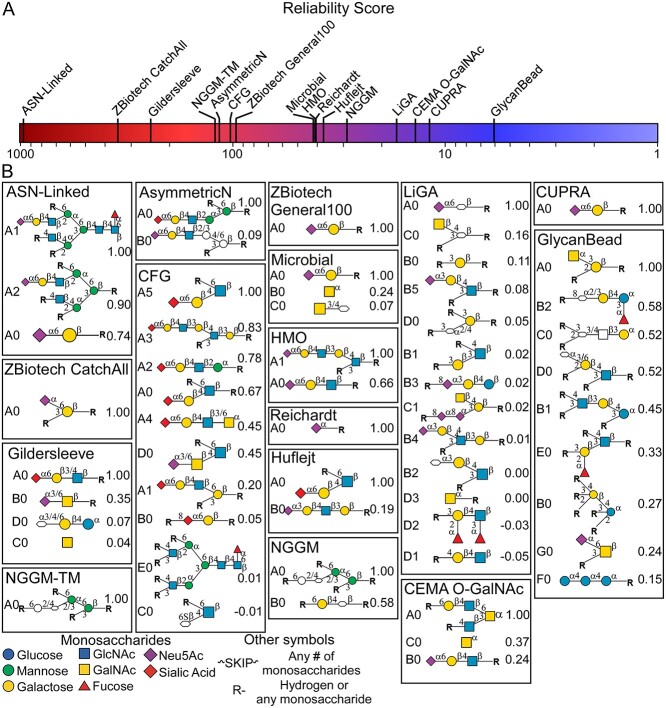
Comparison across multiple arrays of results for SNA. A) Reliability score for each of the arrays, where higher scores indicate lower dataset noise. B) CarboGrove reported motifs for each array. Within each array, the motifs are ordered from top to bottom by the relative-binding score given next to the ID and graphical representation of the motif. The monosaccharide symbols follow the SNFG (Symbol Nomenclature for Glycans) system (PMID 26543186, Glycobiology 25: 1323–1324, 2015) details at NCBI.

We tested the search and analysis capabilities using the lectin SNA (*Sambucus nigra* agglutinin). The primary specificity of SNA, α2,6-linked sialic acids, is well known, but the fine specificities are not well understood owing to limited variety in glycans containing α2,6-linked sialic acid on the arrays and the complexity in the analysis. A search for SNA returned 36 individual datasets from 16 sources (Fig. 4). The datasets had widely varying ranges of dataset noise, as assessed by the reliability score (Fig. 4A), but nearly every data confirmed the canonical specificity of SNA. The top motifs for each array also revealed complementary information. The ASN-linked and AsymmetricN arrays, which focus on N-linked glycans, identified a preference for the tri/tetra-antennary presentation (motifs A1 and A2, ASN-linked array) over the biantennary presentation (motif A0, ASN-Linked array), as well as a preference for the 3′ mannose branch (motif A0, AsymmetricN array) over the 6′ mannose branch or unbranched presentations (motif B0, AsymmetricN array). The CFG array, which has the greatest diversity in the α2,6-sialyl-LacNAc motif, identified preferential binding on extended N-linked glycans over O-linked glycans (motifs A0 and A4). Some arrays had limited variation in the α2,6-sialyl epitope and consequently produced ambiguous, incomplete motifs, such as motifs B0 and D0 on the Gildersleeve array and A0 and the NGGM-TM, NGGM, and Chemoenzymatic Modular Assembly O-GalNAc arrays. Arrays with a low reliability score, such as the LiGA and GlycanBead arrays, generated various weak motifs that are inconsistent with results from other arrays.

We also tested this functionality on the lectin wheat germ agglutinin, which is widely used but has a specificity that is poorly understood. Part of the challenge is its breadth in specificity, binding nearly half of the glycans on the CFG array (273/609 glycans) when applied at high concentrations. The search gave results from 12 different arrays (Supplementary Fig. 1). A comparative analysis identified both known and novel features, such as highest binding to 6′-linked terminal *N*-acetyl-glucosamine (GlcNAc) and 3′-linked Neu5Ac; binding to both GlcNAc and *N*-acetyl galactosamine (GalNAc) in other terminal linkages, provided the 3′ carbon is unsubstituted; and potential binding to the heparan sulfate motif GlcNAca1-4GlcA. The novel observations would require experimental confirmation, but they are structurally plausible and demonstrate findings that are made possible through broad analyses of glycan-array data.

### Selecting glycan-binding proteins for experimental design

**Fig. 5:**
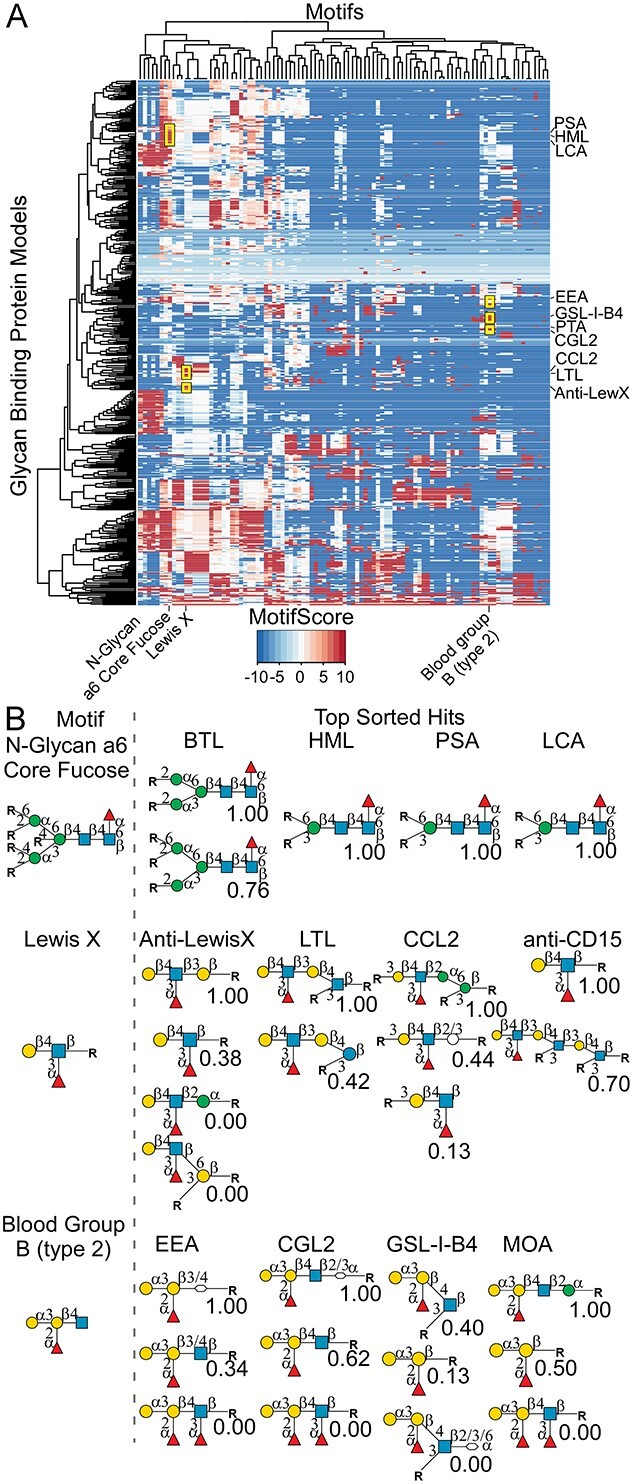
Searches for glycan-binding proteins that are specific for selected motifs. A) The motif scores for 118 motifs (listed in the columns) and 785 glycan-binding proteins were hierarchically clustered. The search motifs and top hits are labeled. B) For the top 4 glycan-binding proteins from each search, the proteins’ top motifs and their relative binding scores are indicated. Monosaccharide symbols follow the SNFG (Symbol Nomenclature for Glycans) system (PMID 26543186, Glycobiology 25: 1323–1324, 2015) details at NCBI.

A companion capability is to select a motif from the motif-sort options and search for glycan-binding proteins that bind the motif. We selected 3 motifs for a test of this function: N-glycan core fucose, Lewis X, and type-2 blood group B. These motifs have the common feature of fucose, but they differ in the fucose linkage: either to the 6′, 3′, or 2′ carbon of the adjoining monosaccharide. A hierarchical cluster of all the models in the database and the set of motifs defined above indicated that the search motifs are bound by separate groups of proteins (Fig. 5A). The top 10 glycan-binding proteins for each motif confirmed that each motif returned a unique set of proteins known to bind the motif (Table 1). An assessment of the top motifs bound by the glycan-binding proteins showed that each protein is a specific binder of the search motif (Fig. 5B).

A closer analysis showed the value of an unbiased search. For example, the top hits for motif “N-Glycan a6 Core Fucose” did not include the lectin commonly used for this motif, *Lens Culinaris* Agglutinin (LCA), because LCA bound many *N*-glycans without alpha-6 fucose (as shown in the CarboGrove model, not shown). The Lewis X and blood group B searches likewise returned results that were unexpected but potentially useful, such as the strong binding to Lewis X of both the anti-Lewis X antibody and the lectins LTA, CCL2, and AAL.

This functionality suggested an additional opportunity in experimental design. Searches such as demonstrated above could be modified to optimally select glycan-binding proteins for an experiment, such as to distinguish between motifs in a biological sample that are difficult to distinguish by mass spectrometry. We tested this concept for terminal GalNAc, in either the alpha or beta orientation, and terminal GlcNAc, in either the bisecting or outer-arm position. These features are isomers but have important differences in biological function.

**Fig. 6:**
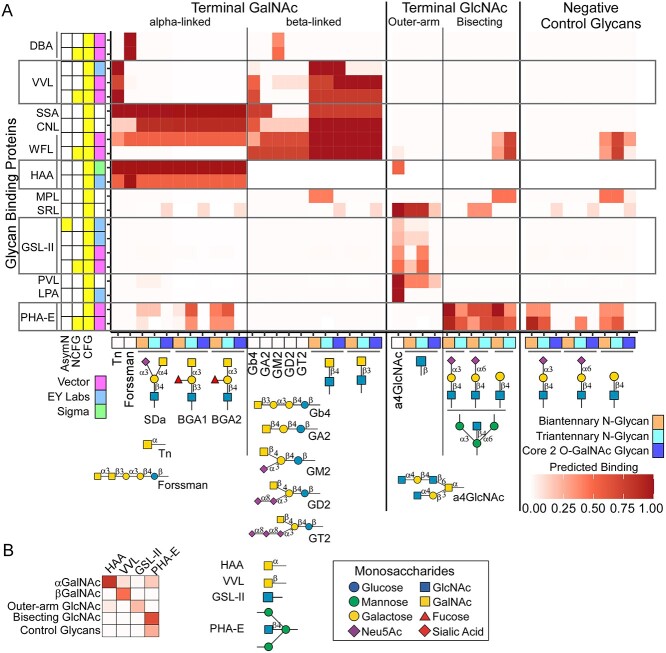
Searches for glycan-binding proteins that are specific for selected motifs. A) Using models downloaded from CarboGrove that bind any of the comparison motifs, we predicted binding to a series of glycans generated in MotifFinder that contain the motifs, as well as negative-control glycans. B) In the reduced set of 4 lectins (GSL-II, HAA, VVL, and PHA-E), the average binding to the glycans in each group shows a different pattern for each group. The differences in top motifs for the lectins correspond to the differences in binding patterns. Monosaccharide symbols follow the SNFG (Symbol Nomenclature for Glycans) system (PMID 26543186, Glycobiology 25: 1323–1324, 2015) details at NCBI.

We sought to identify a minimal set of lectins (limited to 3–4) that would give optimal distinction between the comparison motifs. First, for each of the 4 terminal features (alpha-GalNAc, beta-GalNAc, outer-arm GlcNAc, and bisecting GlcNAc), we defined glycans containing the feature (Fig. 6A). We also defined negative-control glycans that have the core structures but not the terminal features. Next, we searched the database to identify lectins that bind any of the motifs. We predicted the binding of each lectin to each glycan and assembled the values (Fig. 6A), from which we could search for combinations of lectins that give unique patterns of binding across each of the comparison motifs.

Multiple algorithms are available for maximizing distances between subsets. For demonstration, we used manually guided optimization to arrive at the minimal set of GSL-II, HAA, VVL, and PHA-E (Fig. 6B). The average binding of the lectins to the glycans in the comparison groups showed distinct patterns, corresponding to the differences in the top motifs (Fig. 6B): GSL-II binds non-bisecting terminal GlcNAc; HAA binds terminal alpha-GalNAc; VVL binds LacDiNAc and some lipid-linked glycans; and PHA-E binds bisecting GlcNAc. Thus, starting from the full collection of >700 models, we efficiently reduced to just 4 that provide clear distinctions among the 4 isomeric motifs.

## Discussion

The proliferation of glycan-array platforms and data has precipitated a need for a common mode of analyzing, interpreting, and accessing the data. Here we provide a solution via the MotifFinder analysis program and the CarboGrove database. We populated the database with analyzed data from 35 types of arrays and from multiple suppliers and platforms. Thus, for the first time, researchers can access and use an expansive collection of glycan-array data in an analyzed form. The ability to bring together data from multiple sources is especially important in the case of glycan arrays, where each type of array provides information or experiments that are complementary to the others. In particular, the arrays are complementary in their glycans, the glycan-binding proteins analyzed, and the strengths and weaknesses of their experimental systems. The enabling component of this project was a software tool for analyzing all types of glycan-array data. Without a common model of analyzing and reporting the data, the assembly and integrated analysis of such diverse data would be prohibitively time-consuming and inaccurate.

The ability to compare and integrate results between separate datasets has advantages for many applications. In the evaluation of SNA, for which the search returned 36 datasets, we observed fine specificities that are distinct from the canonical specificity and that would be missed in the evaluation of only a single platform. The findings were consistent with the previous studies of SNA. For example, the MotifFinder identification of preferential binding to the primary epitope on extended N-linked glycans from the 3′ mannose branch agrees with the manual analysis (Li, Guan, et al. 2019a). But the ability to conveniently supplement the study with comparable findings from other arrays, such as the preference for tri/tetra-antennary *N*-glycans over linear presentations (Fig. 4), is unique.

These analyses also expand on previous methods to compare between platforms. Previous studies to compare between conditions or platforms generally used manual analyses, such as comparisons between arrays that focused on sialic acids (Padler-Karavani et al. 2012) or between experimental conditions (Temme et al. 2019; Li, Guan, et al. 2019a). An algorithm-based approach was introduced in a well-designed study to compare results between separate array platforms (Wang et al. 2014). The authors used a universal thresholding method to identify differences between platforms that correlated with experimental differences. The method did not, however, capture both weak and strong binding for all arrays and did not account for fact that no single concentration was relevant in comparing results between arrays. In contrast, the algorithm used in the present work is sensitive to weakly bound glycans and allows comparisons among arrays even where concentrations are not optimally matched. Furthermore, the software has the unique support of combined analyses of multiple datasets, previously demonstrated for integrating data from multiple concentrations of a lectin or from multiple platforms (Klamer and Haab 2021).

**Table 1:** Top 10 glycan-binding proteins for each search motif.

Motif	Rank	Lectin	Name	Canonical motif	Source	Array
*N*-Glycan Core a6 Fucose	1	BTL	Bryothamnion Triquetrum Lectin		Investigator	CFG
	2	HML	*Hypnea Musciformis* Lectin		Investigator	CFG
	3	PSA	*Pisum Sativum* Agglutinin	Core fucose	EY Labs	CFG
	4	PSA	*Pisum Sativum* Agglutinin	Core fucose	Vector	CFG
	5	rBTL	Bryothamnion Triquetrum Lectin		Investigator	CFG
	6	PSA	*Pisum Sativum* Agglutinin	Core fucose	Vector	ASNLinked
	7	LCA	*Lens Culinaris* Agglutinin	Core fucose	Vector	ASNLinked
	8	LCA	*Lens Culinaris* Agglutinin	Core fucose	Vector	CFG
	9	LCA	*Lens Culinaris* Agglutinin	Core fucose	Vector	CFG,NCFG
	10	PSA	*Pisum Sativum* Agglutinin	Core fucose	Vector	ZBiotech
Lewis X	1	Anti-LewX	Clone 28 Anti-Lewis X Antibody	Lewis X	Investigator	CFG
	2	LTL	*Lotus Tetragonolobus* Lectin	Fucose	Vector	PolyLacNAc
	3	CCL2	Coprinopsis Cinerea Lectin 2	Fucose alpha1,3	Investigator	CFG
	4	anti-CD15	anti-CD15	Lewis X	Investigator	PolyLacNAc
	5	AAL	Aleuria Aurantia Lectin	Fucose	Vector	PolyLacNAc
	6	CCL2	Coprinopsis Cinerea Lectin 2	Fucose alpha1,3	Investigator	CFG
	7	AAL	Aleuria Aurantia Lectin	Fucose	Vector	FeiziCLL
	8	LTL	*Lotus Tetragonolobus* Lectin	Fucose	Vector	Zbiotech
	9	AAL	Aleuria Aurantia Lectin	Fucose	Vector	CEMAOGalNAc
	10	AAL	Aleuria Aurantia Lectin	Fucose	Vector	AsymN
Blood Group B (type 2)	1	EEA	*Euonymus Europaeus* Agglutinin	Blood group B	EY Labs	CFG
	2	EEA	*Euonymus Europaeus* Agglutinin	Blood group B	Vector	GlycanBead
	3	CGL2	Coprinopsis Cinerea Galectin 2	Fucose alpha1,2	Investigator	CFG
	4	GSL-I-B4	Griffonia Simplicifolia Lectin 1, B4	Galactose	Vector	CFG
	5	MOA	Marasmium Oreades Agglutinin	Alpha-galactose	EY Labs	CFG
	6	PTA	*Psophocarpus Tetragonolobus* Agglutinin	Blood groups	EY Labs	CFG
	7	GSL-I-B4	Griffonia Simplicifolia Lectin 1, B4	Galactose	Vector	CFG,NCFG
	8	EEA	*Euonymus Europaeus* Agglutinin	Blood group B	Vector	CFG
	9	PA-IL	*Pseudomonas Aeruginosa* Lectin 1	Galactose	Sigma	CFG
	10	GSL-I-B4	Griffonia Simplicifolia Lectin 1, B4	Galactose	Vector	Zbiotech

This study also highlighted the need for a standardized system of reporting the details of glycan array experiments. The MIRAGE guidelines for reporting glycan-array metadata (Supplementary Table II) help to improve accessibility of metadata and the ability to compare results (Liu et al. 2017). Tools designed to standardize the design, processing, and storage of glycan array data like the in-development CarbArrayArt and GlyGen glycan array database will further improve the accessibility of glycan array metadata (Mehta et al. 2020).

Besides studying the specificities of glycan-binding proteins, a major function of CarboGrove is to identify glycan-binding proteins that have user-specified binding traits. This function will be important for both non-experts and experts in glycobiology. Searches across lesser-known platforms and motifs can be impractical, and the specificities of some glycan-binding proteins are not generally known or are misunderstood. But even more valuable could be the ability to select optimal sets of lectins for experiments. The use of lectins to detect or quantify glycan structures is very common, for example in methods such as immunofluorescence, western blot, cell staining, in vivo imaging, and others, but in many cases, the experiments do not employ the optimal lectins or are inaccurately interpreted. Using CarboGrove, a researcher could perform searches to identify a limited number of glycan-binding proteins that target the motifs that are relevant to the biological study. MotifFinder could predict binding to a set of relevant glycans and select the proteins most useful for analysis. Bioinformaticians could support this work by developing tools employing additional approaches to optimize experiments that use glycan-binding proteins.

The current study and resource have several limitations. The analysis algorithm does not account for certain features that could influence binding, such as the method of attaching the glycan, the density of the glycan, or the nature of a polypeptide backbone if present. A wide variety of experimental conditions also can influence apparent binding, including buffer, array substrate, and detection method (Kitova et al. 2019). The resource currently does not house much of the metadata associated with the experiments, from which one could explore factors that influence binding. The metadata exists in a great variety of completeness and form, so the inclusion of all available information in the database is a technical hurdle. Currently, the database includes references to the original sources of the data, through which researchers locate complete metadata if needed. While the database does house all the data used in the analyses performed here (accessible via the Application Programming Interface), the database is not intended to be a resource for data deposition, but conceivably such a development could be useful. Finally, enabling support for user-defined motifs for database searches could expand the specificity-finding capabilities of the resource. This addition requires additional developments in the motif-building tools and would dramatically increase the computational overhead of the database and thereby database operating costs, one of the limiting factors in bioinformatics-resource lifecycles.

Another important goal is to provide connections between resources providing complementary information, for example regarding the glycans, the motifs, the GBPs, or the platforms would be valuable. For bioinformatics developers, such connectivity is already available through the Application Programming Interface (API) in CarboGrove. For general users, links to the UniLectin database via the stable UniProt accession numbers of the lectin are currently provided, and links to addition information, such as through GlyTouCan or GlyGen, will be continuously added in updates (Bonnardel et al. 2019; York et al. 2019; Consortium et al. 2020; Fujita et al. 2020). Many additional developments in bioinformatics could be stimulated by this resource. For example, bioinformaticians could use the resource to explore relationships among families of lectins and antibodies in association with genetic or organismal information. The motifs from MotifFinder could be used as the connection to a wide range of data on sequence, biosynthesis, and other information, such as are accessible through the GlyGen resource (York et al. 2019) and other databases. A resource that appeared after submission of this work is a machine learning annotation of lectin specificities, which could provide additional, complementary information (Bojar et al. 2022).

The pace of introduction of new arrays and glycans is clearly quickening. Versatile systems of attachment to surfaces using both covalent and noncovalent deposition (Li et al. 2021) could make array production easier for nonspecialist labs. Others have displayed glycans on the surface of bacteriophage (Sojitra et al. 2021), produced various glycans through sequential knockdowns of genes in glycan biosynthesis (Büll et al. 2021), and tuned the density of glycans spotted on chip through efficient methods of producing glycopeptide arrays (Mende et al. 2020). The MotifFinder platform supports updating to allow for additional factors to be explored. Our ongoing work involves support for investigating the influence of peptide backbone, glycan density, and linker type, as well as experimental factors that have been shown to introduce variability (Temme et al. 2019). Thus, we present a system to handle the additional types of information as glycan arrays continue to expand into new areas.

## Methods

### Data collection

The collaborators at Z Biotech provided 151 glycan-array datasets generated for internal quality control studies, including data for 43 different glycan-binding proteins collected on 11 of the arrays offered by the company. Raw data are available as supplementary data (Supplementary Table III). Details on the data collection are provided in the supplementary MIRAGE document (Supplementary Table II).

Data for the CFG and NCFG arrays were retrieved from their respective websites and databases. Data from individual laboratories were retrieved from the original publications or provided by the authors upon request.

### Statistical analysis

The prediction of binding to glycans using the models was described previously (Klamer and Haab 2021) and is detailed in the user’s manual provided with MotifFinder. The reliability score, which was used to rank models by the quality of their results (Fig. 4), measures the difference between the average binding of the top motif and the average binding of the non-binding motifs, normalized by the standard deviation in the non-binding motifs. This metric is similar to the signal-to-noise ratio. Given the average of the top-binding glycan values *m*, the average of the non-binding glycan values *v*, the standard deviation of the non-binding glycan values *s*, and the number of datasets in the model *n*, the reliability score is calculated as:

$$ \mathrm{Reliability}\ \mathrm{score}=\frac{\sum_1^n\left(\max \left({m}_n\right)-{v}_n\right)/{s}_n}{n} $$

### Data analysis

The majority of datasets could be analyzed as they were provided, with minor corrections in text syntax to match the modified IUPAC syntax used by the CFG. In one case, the CUPRA array, the data needed to be inverted to enforce the requirement of a positive association between binding and the quantification of the binding. All data were analyzed using MotifFinder release version V2.2.5 (Klamer and Haab 2020).

The Motif Score was calculated as described previously (Klamer et al. 2017). Briefly, a *t*-test with unequal variances is performed comparing the predicted binding of glycans with the motif to those without the motif. The resulting *P*-value is log10 transformed and re-signed to match the sign of the *t* value from the *t*-test. Motif Score values are truncated to the range of −10 to 10. The Motif Score is used to rank associations with binding rather than for statistics. Additional metrics are used to break ties in the Motif Score for motif sorting, including the average binding of glycans with the motif and the precision of the motif (the number of glycans with the motif that has a positive predicted binding divided by the number of glycans with the motif).

### Assigning standardized IDs to database contents

The assignment of UniProt IDs and PFAM families was done manually through searching the UniProt database (accessed 2022 July 2) (Consortium et al. 2020). Assignment of GlyTouCan IDs to glycans (accessible via the API as part of the get_data and get_result functions) required several steps. MotifFinder’s built-in parser is capable of parsing glycans in their raw format (CFG-like IUPAC condensed). We adapted MotifFinder’s glycan printing function to print the standardized IUPAC condensed format for all parsable glycans. The IUPAC condensed format was passed to the GlycanFormatConverter tool to convert the glycans to WURCS glycan format (Tsuchiya et al. 2019). The GlyCosmos portal offers GlyTouCan search by text to get GlyTouCan IDs (Yamada et al. 2020).

### Database Design and Programmatic Access

The database was developed using MariaDB (an open-source MySQL branch) and delivered using php (version 7.1.28) for server-side processing of searches and database interface. Javascript was used for the web interface and the compression of webserver file uploads. Bash scripts and the jq json parser tool were used to process, manage, and return webserver requests. The database and webserver are hosted using Amazon Web Services. An overview of the data ingestion process and API data delivery is given in Supplementary Fig. 2. Briefly, information about the glycan-binding protein, the experimental system, and the data are parsed and entered into multiple tables. The raw data and the results from the MotifFinder analyses are linked to this information.

Programmatic access to the CarboGrove database is served through an API that allows bioinformatics users to access the database in computational formats and utilize the curated list of glycan binding protein aliases to standardize glycan binding protein names. Details on the use of the API are given in the API page in the CarboGrove website, and a high-level overview of the data-retrieval process is given in Supplementary Fig. 2.

## Author contributions

Conceptualization: ZLK, BBH.

Data curation and software: ZLK.

Investigation: CMH, JMB, JEK.

Visualization: ZLK, BBH.

Supervision: BBH, JZ.

Writing—original draft: BBH, ZLK.

Writing—review and editing: BBH, ZLK.

## Supplementary Material

CarboGrove_Glycobiology_supplementary_v3_1_Revision_cwac022

Supplementary_Table_III_cwac022

Supplementary_Table_IV-VI_DatabaseStats_Revision_cwac022

CarboGrove_SupplementalFigure1_cwac022

SupplementalFigure_S2_cwac022

## Data Availability

The models reported here are available through the CarboGrove website. MotifFinder is available as a standalone tool or through a webserver. The webserver features a simplified interface while running the same algorithm as the standalone program. CarboGrove is licensed under the CC BY-SA 4.0 license and can be accessed at https://carbogrove.org/. Raw glycan array data for published datasets are available from their respective publications, and processed data are available through the API or via download in bulk from FigShare: https://figshare.com/articles/dataset/CarboGrove_Glycan_Binding_Data/19274777. Details on the use of CarboGrove API are available under the API homepage: https://carbogrove.org/api/home.php. Details on the use of CarboGrove and use cases for CarboGrove and its API are available through the help page: https://carbogrove.org/help.php. Unpublished glycan-array data are available in the supplementary materials. The compiled MotifFinder software used to generate all results in CarboGrove is freely available for academic, nonprofit, and research use through our download page (https://haablab.vai.org/tools/).

## Abbreviations

AAL, *Aleuria Aurantia* Lectin; API, Application Programming Interface; CCL2, *Coprinopsis Cinerea* Lectin 2; CFG, Consortium for Functional Glycomics; CEMA, Chemoenzymatic Modular Assembly; GalNAc, N-acetyl galactosamine; GlcA, glucuronic acid; GlcNAc, N-acetyl glucosamine; GlyMDB, Glycan Microarray Database; GLAD, Glycan Array Dashboard; GSL-II, *Griffonia Simplicifolia* Lectin 2; HAA, *Helix Aspersa* Agglutinin; LacDiNAc, GalNAc, GlcNAc disaccharide; LacNAc, Galactose, GlcNAc disaccharide; LiGA, Liquid Glycan Array; LCA, *Lens Culinaris* Agglutinin; LFDB, Lectin Frontier Database; LTA, *Lotus Tetragonolobus* Lectin; MCAW-DB, Multiple Carbohydrate Alignment with Weights Database; PHA-E, *Phaseolus Vulgaris* Erythroagglutinin; Neu5Ac, 5-acetyl neuramic acid; Neu5Gc, 5-glycolyl neuramicacid; NCFG, National Center for Functional Glycomics; NGGM, Next Generation Glycan Microarray; NGGM-TM, Next Generation Glycan Microarray Traditional Microarray Comparison; SNA, *Sambucus Nigra* Agglutinin 1; WGA, Wheat Germ Agglutinin
