# Supplementary material for: CarboGrove: a resource of glycan-binding specificities through analyzed glycan-array datasets from all platforms
Source: Glycobiology. 2022 Mar 29;32(8):679–90. doi: 10.1093/glycob/cwac022 (PMC9280547; doi:10.1093/glycob/cwac022)
Supplement: CarboGrove_Glycobiology_supplementary_v3_1_Revision_cwac022 [file carbogrove_glycobiology_supplementary_v3_1_revision_cwac022.docx]

Supplementary Materials for

**CarboGrove: a resource of glycan-binding specificities through analyzed glycan-array datasets from all platforms**

Zachary L. Klamer, Chelsea M. Harris, Jonathan M. Beirne, Jessica E. Kelly, Jian Zhang, and Brian B. Haab*

*Corresponding author: Brian B. Haab, [brian.haab@vai.org](mailto:brian.haab@vai.org)

**This PDF file includes:**

Figures S1 and S2

Tables SI to SII

**Other Supplementary Materials for this manuscript include the following:**

Tables SIII to SVI (Excel files)

Supplementary Figures


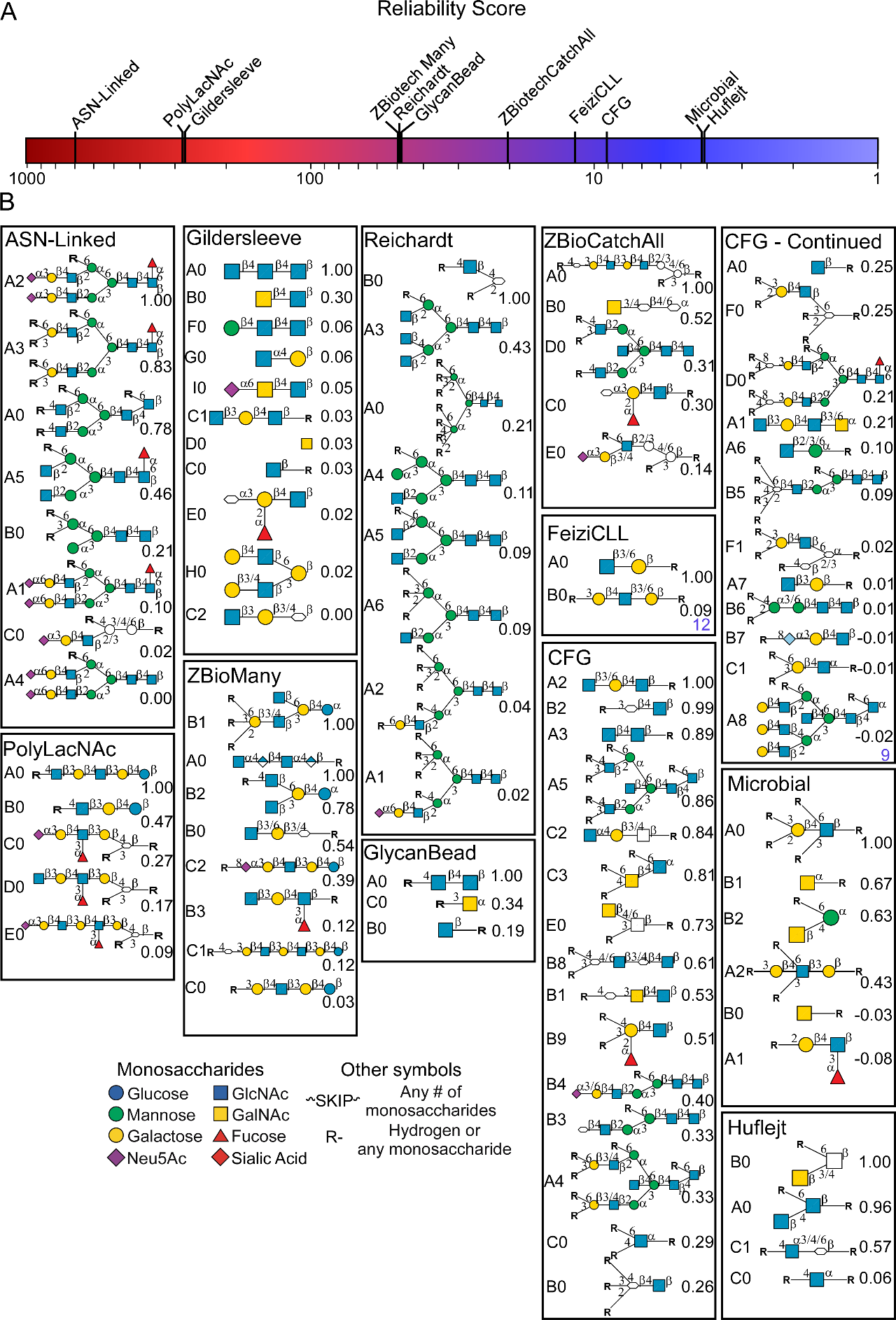


**Figure S1.** **Comparison across multiple arrays of results for WGA.** (A) The calculated reliability score for each of the arrays, where reliability score is an indicator of dataset noise. (B) CarboGrove reported motifs for each array. The relative-binding score is given next to the ID and graphical representation of each motif. The consensus between arrays indicates a general binding to N-acetyl containing (NAc) monosaccharides with sensitivity to the presentation of the epitope. While the NAc-monosaccharide does not need to be terminal, no array indicates binding to 3’ substituted NAc-monosaccharides. The highest binding seems to occur for 6’ presented HexNAc structures or 3’ presented Neu5Ac structures, which is consistent with the shift in the position of the N-acetyl group from the 2’ carbon to the 5’ carbon. In addition, the ZBioMany data indicating binding to the heparan sulfate motif GlcNAca1-4GlcA. Monosaccharide symbols follow the SNFG (Symbol Nomenclature for Glycans) system ([PMID 26543186](https://pubmed.ncbi.nlm.nih.gov/26543186/), Glycobiology 25: 1323–1324, 2015) details at [NCBI](https://www.ncbi.nlm.nih.gov/books/NBK310273/)

**
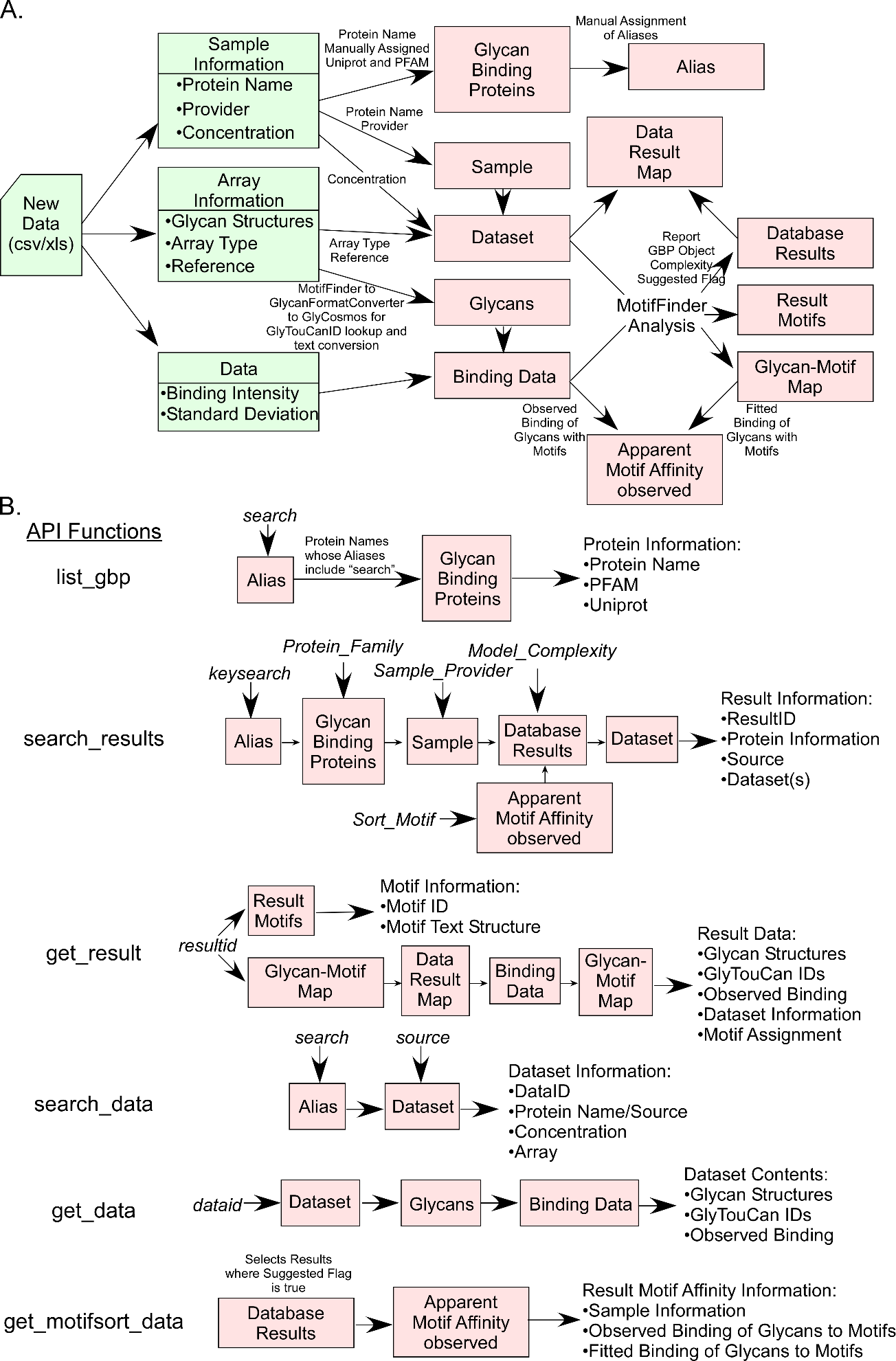
**

**Figure S2.** **Data Ingestion and API Access Diagrams.** (A) As new data are added to the database, several tables are populated. A new entry is added to the Glycan-Binding Protein table and the Sample table for the specific protein and source used. For new arrays, the glycan names are converted to exchange formats and added to the list of all glycans. For each dataset, a data ID is given and the binding data are stored. The results are stored from the several MotifFinder analyses that may be run at different parameters for one or more of these datasets. (B) Using the 6 available API functions, bioinformatics users can take advantage of the extensive list of aliases to retrieve unique identifiers for glycan-binding proteins with relevant cross-reference identifiers, where available. Users also can search or retrieve both results and data, with search_results utilizing the same search parameters as the web version of the database. Finally, users can retrieve the data utilized in the motif sort capabilities of the CarboGrove search function. This information provides a useful summary of the apparent affinity of glycan-binding proteins to multiple predefined motifs.

**Supplementary Tables**

Supplementary Table I. Glycan Array Timeline and References

| Milestone | Publication Date |
| --- | --- |
| CFG Formed | 09/01 |
| Synthetic Neoglycolipid Array(Chai et al. 2003) | 12/03 |
| Paulson Mammalian Array(Blixt et al. 2004) | 10/04 |
| Gildersleeve Neoglycoprotein Array(Manimala et al. 2006) | 05/06 |
| Mannose 6P Array(Song et al. 2009) | 12/09 |
| Modified Sialic Acid Array(Song et al. 2011) | 07/11 |
| Schistosma Glycan Array(Mickum et al. 2016) | 04/16 |
| Human Milk Oligosaccharide Array(Prudden et al. 2017) | 07/17 |
| Bulky Asymmetric N-Glycan Array(Wu et al. 2017) | 10/17 |
| Plant Cell Wall Array(Ruprecht et al. 2017) | 11/17 |
| Glycan Bead Array(Purohit et al. 2018) | 01/18 |
| Wang Automated Synthesis(Zhang et al. 2018) | 10/18 |
| Chemoenzymatic N-Glycan Array(Liu et al. 2019) | 12/18 |
| Boons Automated Synthesis(T. Li et al. 2019) | 03/19 |
| ASN-Linked N-Glycan Array(Gao et al. 2019) | 04/19 |
| Synthetic Microbial Glycan Array(Geissner et al. 2019) | 05/19 |
| Next-Gen Glycan Microarray (NGGM)(Yan et al. 2019) | 06/19 |
| Competitive Universal Proxy Receptor Assay Array (CUPRA)(Kitov et al. 2019) | 07/19 |
| Beam Search Array/Feizi CLL Array(Wu et al. 2019) | 10/19 |
| Sialoglycan Neoglycolipid Array(Murugesan et al. 2020) | 05/20 |
| Asymmetric N-Glycan Array(L. Li et al. 2019) | 05/20 |
| Poly-LacNAc Glycan Array(Chen et al. 2020) | 06/20 |
| Liu Heparan Sulfate Array(Horton et al. 2020) | 06/20 |
| Boons Heparan Sulfate Array(Chopra et al. 2021) | 01/21 |
| Liquid Glycan Array (LiGA)(Sojitra et al. 2021) | 05/21 |
| Chemo-enzymatic Microarray O-GalNAc Glycan Array (CEMA O-GalNAc)(Wang et al. 2021) | 06/21 |
| Oligomannose Glycan Array(Gao et al. 2021) | 06/21 |

Supplementary Table II. Glycan Array MIRAGE Guidelines. The table provides the standard information specified by the MIRAGE guidelines. The unpublished data in Supplemental Table 3, provided by Z Biotech, were collected under these guidelines

|  | | | **Description** |
| --- | --- | --- | --- |
| 1. **Sample: Glycan Binding Sample** | | | |
| Description of Sample | Glycan binding protein name, source, and concentration described in the supplemental data file. | | |
| Sample modifications | All lectins were biotinylated or AF555 labeled as noted in data file. | | |
| Assay protocol | Each assay was performed following the assay protocol laid out in the user’s manual found for each array on ZBiotech’s website (<http://www.zbiotech.com/products.html>). | | |
| **2.** **Glycan Library** | | | |
| Glycan description for defined glycans | 1. Heparan Sulfate glycans were purchased from Glycan Therapeutics or Iduron. 2. Glycans on the Neu5Gc/Neu5Ac N-glycans were synthesized under a NIH SBIR grant (GM123820). 3. Glycans on the HMO Glycan Array were synthesized under a NIH SBIR grant (GM123820). 4. Glycans on the O-mannose glycans were synthesized under a NIH SBIR grant (GM123820). 5. GSL glycans were purchased from Glycohub, Inc.and Elicityl SA. They were further purified by HPLC to meet the purity >95%. 6. O-glycans were synthesized under a NIH SBIR Grant (GM123820). 7. N-glycans were synthesized under a NIH SBIR Grant (GM123820). | | |
| Glycan description for undefined glycans | No glycan is undefined | | |
| Glycan modifications | 1. Heparan sulfate glycans were in the form of free-reducing end (no modification). 2. Glycans on the Neu5Gc/Neu5Ac N-glycans were in the form of free-reducing end (no modification). 3. Glycans on the HMO Glycan Array were modified with a proprietary amino tag at the reducing end. 4. Glycans on the O-mannose glycans were modified by a threonine tag at the reducing end. 5. The GSL glycans were modified with a proprietary amino tag at the reducing end. 6. The O-glycans were modified by a serine or threonine tag at the reducing end. 7. N-glycans were in the form of free reducing-end (no modification). | | |
| 1. **3.** **Printing Surface; e.g., Microarray Slide** | | | |
| Description of surface | 1. The General 100 Glycan Array, Heparan Sulfate Glycan Array, Neu5Gc/Neu5Ac N-glycan Array, and general N-glycan arrary were fabricated on a hydrazide functionalized microarray substrate. 2. The Catch-all Array, Bisecting N-glycan Array, HMO Glycan Array, O-glycan Array, GSL Glycan Array, and O-Mannose Glycan Array were fabricated on the NHS-ester functionalized microarray substrate. | | |
| Manufacturer | Z Biotech, LLC  10501-3: Multivalent Hydrazide Slides (for General 100 Glycan Array, Heparan Sulfate Glycan Array, Neu5Gc/Neu5Ac N-glycan Array, and general N-glycan array)  10401-3: Multivalent NHS Slides (for Catch-all Array, Bisecting N-glycan Array, HMO Glycan Array, O-glycan Array, and O-Mannose Glycan Array) | | |
| Custom preparation of surface | None | | |
| Non-covalent Immobilization | None. All glycans were covalently immobilized onto the microarray substrates. | | |
| **4. Arrayer (Printer)** | | | |
| Description of Arrayer | sciFLEXARRAYER S3 (Scienion) | | |
| Dispensing mechanism | No-contact dispensing | | |
| Glycan deposition | Each glycan was deposited ~1.2 nL per spot. Each slide contains 8- or 16-subarray. Each subarray contains at least 3 replicate spots for each glycan. | | |
| Printing conditions | For printing Catch-all Array, Bisecting N-glycan Array, HMO Glycan Array, GSL, O-glycan Array and O-Mannose Glycan Array, glycans were dissolved in 150 mM sodium phosphate buffer (pH 8.5) at 100 uM concentration. For printing General 100 Glycan Array, Heparan Sulfate Glycan Array and Neu5Gc/Neu5Ac N-glycan Array, general N-Glycan Array, glycans were dissolved in 150 mM sodium phosphate buffer (pH 5.8) at 100 uM concentration. The glycans were spotted at ambient temperature and relative humidity 50%. | | |
| 1. **5.** **Glycan Microarray with “Map”** | | | |
| Array layout | | Each array was laid out according to the user manual on Z Biotech’s website (<http://www.zbiotech.com/products.html>). The .gal files were applied during data analysis. | |
| Glycan identification and quality control | | In routine QC process, each batch of glycan array products have been assayed with individual plant lectins (e.g., ConA, AAL, SNA). | |
| 1. **6. Detector and Data Processing** | | | |
| Scanning hardware | Innoscan 710 (Innopsys) | | |
| Scanner settings | Scanning resolution: 10 um / pixel  Laser channel: 532 nm  PMT voltages: Adjust for each sample to achieve decent signal without saturation of any single spot and without high background.  Scan Power: Adjust for each sample to achieve decent signal without saturation of any single spot and without high background. | | |
| Image analysis software | Mapix (Innopsys) | | |
| Data processing | Raw data were output as .gpr files which were converted to excel files. Then data were processed by a data sorting software and a binding motif mining software (MotifFinder). | | |
| **7.** **Glycan Microarray Data Presentation** | | | |
| Data presentation | The Relative Fluorescence Units (RFU) data were presented as bar graphs with error bars representing standard deviation from values of replicate spots. The MotifFinder report example is presented on Z Biotech’s website (<http://www.zbiotech.com/tools.html>). | | |
| 1. **8.** **Interpretation and** **Conclusion from Microarray Data** | | | |
| Data interpretation | MotifFinder software and its running algorithms were used to interpret process data. The development of MotifFinder was supported by a NIH SBIR grant (GM131430). | | |
| Conclusions |  | | |

References

Blixt O, Head S, Mondala T, Scanlan C, Huflejt ME, Alvarez R, Bryan MC, Fazio F, Calarese D, Stevens J, et al. 2004. Printed covalent glycan array for ligand profiling of diverse glycan binding proteins. P Natl Acad Sci Usa. 101(49):17033–17038. doi:10.1073/pnas.0407902101.

Chai W, Stoll MS, Galustian C, Lawson AM, Feizi T. 2003. Neoglycolipid Technology: Deciphering Information Content of Glycome. Methods Enzymol. 362:160–195. doi:10.1016/s0076-6879(03)01012-7.

Chen C, Wang S, Gadi MR, Zhu H, Liu F, Liu C-C, Li L, Wang F, Ling P, Cao H. 2020. Enzymatic modular synthesis and microarray assay of poly- N -acetyllactosamine derivatives. Chem Commun. 56(55):7549–7552. doi:10.1039/d0cc03268a.

Chopra P, Joshi A, Wu J, Lu W, Yadavalli T, Wolfert MA, Shukla D, Zaia J, Boons G-J. 2021. The 3-O-sulfation of heparan sulfate modulates protein binding and lyase degradation. Proc National Acad Sci. 118(3):e2012935118. doi:10.1073/pnas.2012935118.

Gao C, Hanes MS, Byrd-Leotis LA, Wei M, Jia N, Kardish RJ, McKitrick TR, Steinhauer DA, Cummings RD. 2019. Unique Binding Specificities of Proteins toward Isomeric Asparagine-Linked Glycans. Cell Chem Biol. 26(4):535-547.e4. doi:10.1016/j.chembiol.2019.01.002.

Gao C, Stavenhagen K, Eckmair B, McKitrick TR, Mehta AY, Matsumoto Y, McQuillan AM, Hanes MS, Eris D, Baker KJ, et al. 2021. Differential recognition of oligomannose isomers by glycan-binding proteins involved in innate and adaptive immunity. Sci Adv. 7(24):eabf6834. doi:10.1126/sciadv.abf6834.

Geissner A, Reinhardt A, Rademacher C, Johannssen T, Monteiro J, Lepenies B, Thépaut M, Fieschi F, Mrázková J, Wimmerova M, et al. 2019. Microbe-focused glycan array screening platform. Proc National Acad Sci. 116(6):201800853. doi:10.1073/pnas.1800853116.

Horton M, Su G, Yi L, Wang Z, Xu Y, Pagadala V, Zhang F, Zaharoff DA, Pearce K, Linhardt RJ, et al. 2020. Construction of heparan sulfate microarray for investigating the binding of specific saccharide sequences to proteins. Glycobiology. 31(3):188–199. doi:10.1093/glycob/cwaa068.

Kitov PI, Kitova EN, Han L, Li Z, Jung J, Rodrigues E, Hunter CD, Cairo CW, Macauley MS, Klassen JS. 2019. A quantitative, high-throughput method identifies protein–glycan interactions via mass spectrometry. Commun Biology. 2(1):268. doi:10.1038/s42003-019-0507-2.

Li L, Guan W, Zhang G, Wu Z, Yu H, Chen X, Wang PG. 2019. Microarray analyses of closely related glycoforms reveal different accessibilities of glycan determinants on N-glycan branches. Glycobiology. 30(5):334–345. doi:10.1093/glycob/cwz100.

Li T, Liu L, Wei N, Yang J-Y, Chapla DG, Moremen KW, Boons G-J. 2019. An automated platform for the enzyme-mediated assembly of complex oligosaccharides. Nat Chem. 11(3):229–236. doi:10.1038/s41557-019-0219-8.

Liu L, Prudden AR, Capicciotti CJ, Bosman GP, Yang J-Y, Chapla DG, Moremen KW, Boons G-J. 2019. Streamlining the chemoenzymatic synthesis of complex N-glycans by a stop and go strategy. Nat Chem. 11(2):161–169. doi:10.1038/s41557-018-0188-3.

Manimala JC, Roach TA, Li Z, Gildersleeve JC. 2006. High‐Throughput Carbohydrate Microarray Analysis of 24 Lectins. Angew Chem-ger Edit. 118(22):3689–3692. doi:10.1002/ange.200600591.

Mickum ML, Prasanphanich NS, Song X, Dorabawila N, Mandalasi M, Lasanajak Y, Luyai A, Secor WE, Wilkins PP, Die IV, et al. 2016. Identification of Antigenic Glycans from Schistosoma mansoni by Using a Shotgun Egg Glycan Microarray. Infect Immun. 84(5):1371–1386. doi:10.1128/iai.01349-15.

Murugesan G, Correia VG, Palma AS, Chai W, Li C, Feizi T, Martin E, Laux B, Franz A, Fuchs K, et al. 2020. Siglec-15 recognition of sialoglycans on tumor cell lines can occur independently of sialyl Tn antigen expression. Glycobiology. doi:10.1093/glycob/cwaa048.

Prudden AR, Liu L, Capicciotti CJ, Wolfert MA, Wang S, Gao Z, Meng L, Moremen KW, Boons G-J. 2017. Synthesis of asymmetrical multiantennary human milk oligosaccharides. Proc National Acad Sci. 114(27):6954–6959. doi:10.1073/pnas.1701785114.

Purohit S, Li T, Guan W, Song X, Song J, Tian Y, Li L, Sharma A, Dun B, Mysona D, et al. 2018. Multiplex glycan bead array for high throughput and high content analyses of glycan binding proteins. Nat Commun. 9(1):258. doi:10.1038/s41467-017-02747-y.

Ruprecht C, Bartetzko MP, Senf D, Dallabernadina P, Boos I, Andersen MCF, Kotake T, Knox JP, Hahn MG, Clausen MH, et al. 2017. A Synthetic Glycan Microarray Enables Epitope Mapping of Plant Cell Wall Glycan-Directed Antibodies. Plant Physiol. 175(3):1094–1104. doi:10.1104/pp.17.00737.

Sojitra M, Sarkar S, Maghera J, Rodrigues E, Carpenter EJ, Seth S, Vinals DF, Bennett NJ, Reddy R, Khalil A, et al. 2021. Genetically encoded multivalent liquid glycan array displayed on M13 bacteriophage. Nat Chem Biol.:1–11. doi:10.1038/s41589-021-00788-5.

Song X, Lasanajak Y, Olson LJ, Boonen M, Dahms NM, Kornfeld S, Cummings RD, Smith DF. 2009. Glycan Microarray Analysis of P-type Lectins Reveals Distinct Phosphomannose Glycan Recognition. J Biol Chem. 284(50):35201–35214. doi:10.1074/jbc.m109.056119.

Song X, Yu H, Chen X, Lasanajak Y, Tappert MM, Air GM, Tiwari VK, Cao H, Chokhawala HA, Zheng H, et al. 2011. A Sialylated Glycan Microarray Reveals Novel Interactions of Modified Sialic Acids with Proteins and Viruses. J Biol Chem. 286(36):31610–31622. doi:10.1074/jbc.m111.274217.

Wang S, Chen C, Gadi MR, Saikam V, Liu D, Zhu H, Bollag R, Liu K, Chen X, Wang F, et al. 2021. Chemoenzymatic modular assembly of O-GalNAc glycans for functional glycomics. Nat Commun. 12(1):3573. doi:10.1038/s41467-021-23428-x.

Wu N, Silva LM, Liu Y, Zhang Y, Gao C, Zhang F, Fu L, Peng Y, Linhardt R, Kawasaki T, et al. 2019. Glycan Markers of Human Stem Cells Assigned with Beam Search Arrays*[S]. Mol Cell Proteomics. 18(10):1981–2002. doi:10.1074/mcp.ra119.001309.

Wu Z, Liu Y, Li L, Wan X-F, Zhu H, Guo Y, Wei M, Guan W, Wang PG. 2017. Decoding glycan protein interactions by a new class of asymmetric N -glycans. Org Biomol Chem. 15(42):8946–8951. doi:10.1039/c7ob02303k.

Yan M, Zhu Y, Liu X, Lasanajak Y, Xiong J, Lu J, Lin X, Ashline D, Reinhold V, Smith DF, et al. 2019. Next-Generation Glycan Microarray Enabled by DNA-Coded Glycan Library and Next-Generation Sequencing Technology. Anal Chem. 91(14):9221–9228. doi:10.1021/acs.analchem.9b01988.

Zhang J, Chen C, Gadi MR, Gibbons C, Guo Y, Cao X, Edmunds G, Wang S, Liu D, Yu J, et al. 2018. Machine‐Driven Enzymatic Oligosaccharide Synthesis by Using a Peptide Synthesizer. Angewandte Chemie Int Ed. 57(51):16638–16642. doi:10.1002/anie.201810661.
