# Supplementary figures and images for: CarboGrove: a resource of glycan-binding specificities through analyzed glycan-array datasets from all platforms
Source: Glycobiology. 2022 Mar 29;32(8):679–90. doi: 10.1093/glycob/cwac022 (PMC9280547; doi:10.1093/glycob/cwac022)

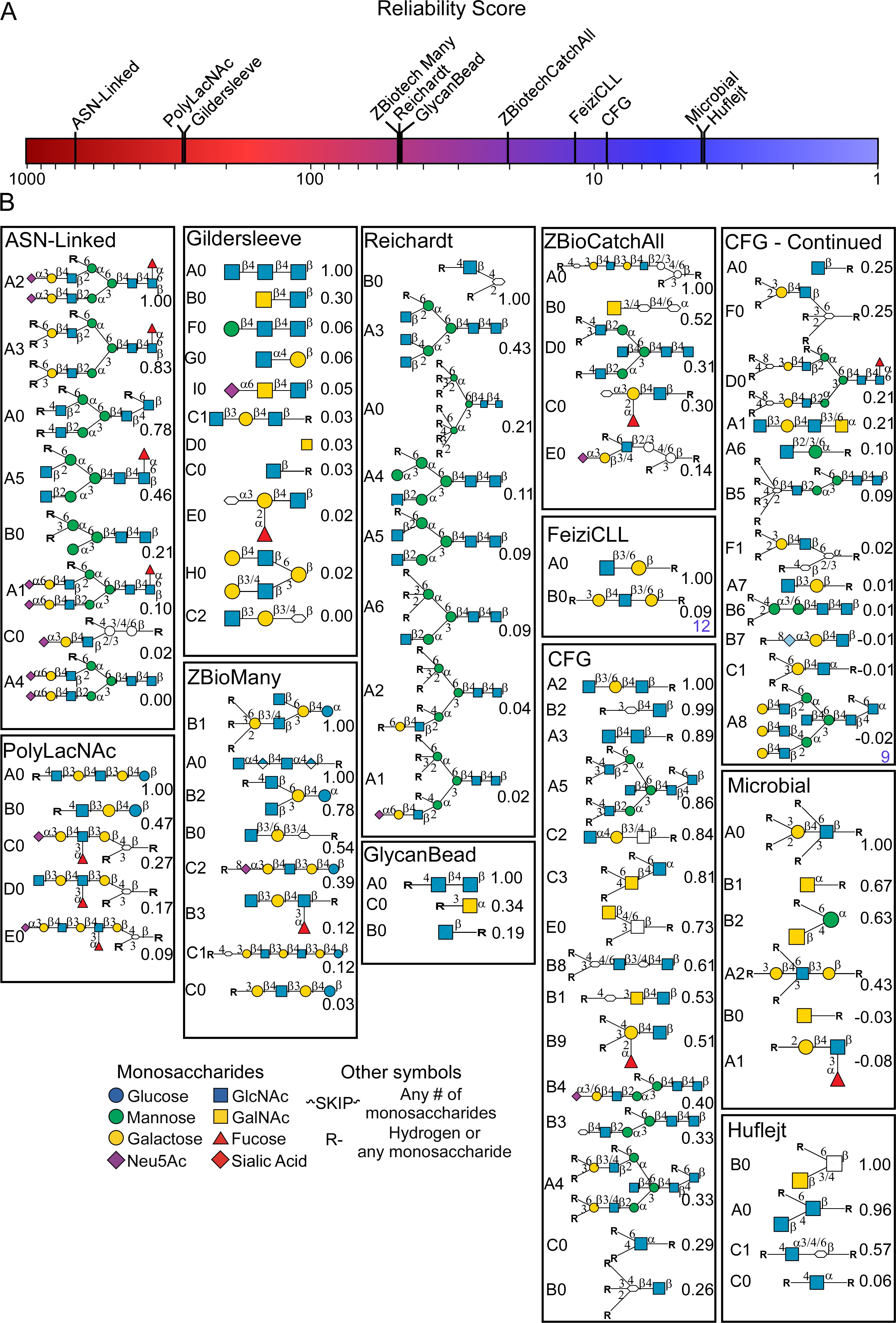

Supplement: CarboGrove_SupplementalFigure1_cwac022 [file carbogrove_supplementalfigure1_cwac022.jpeg]

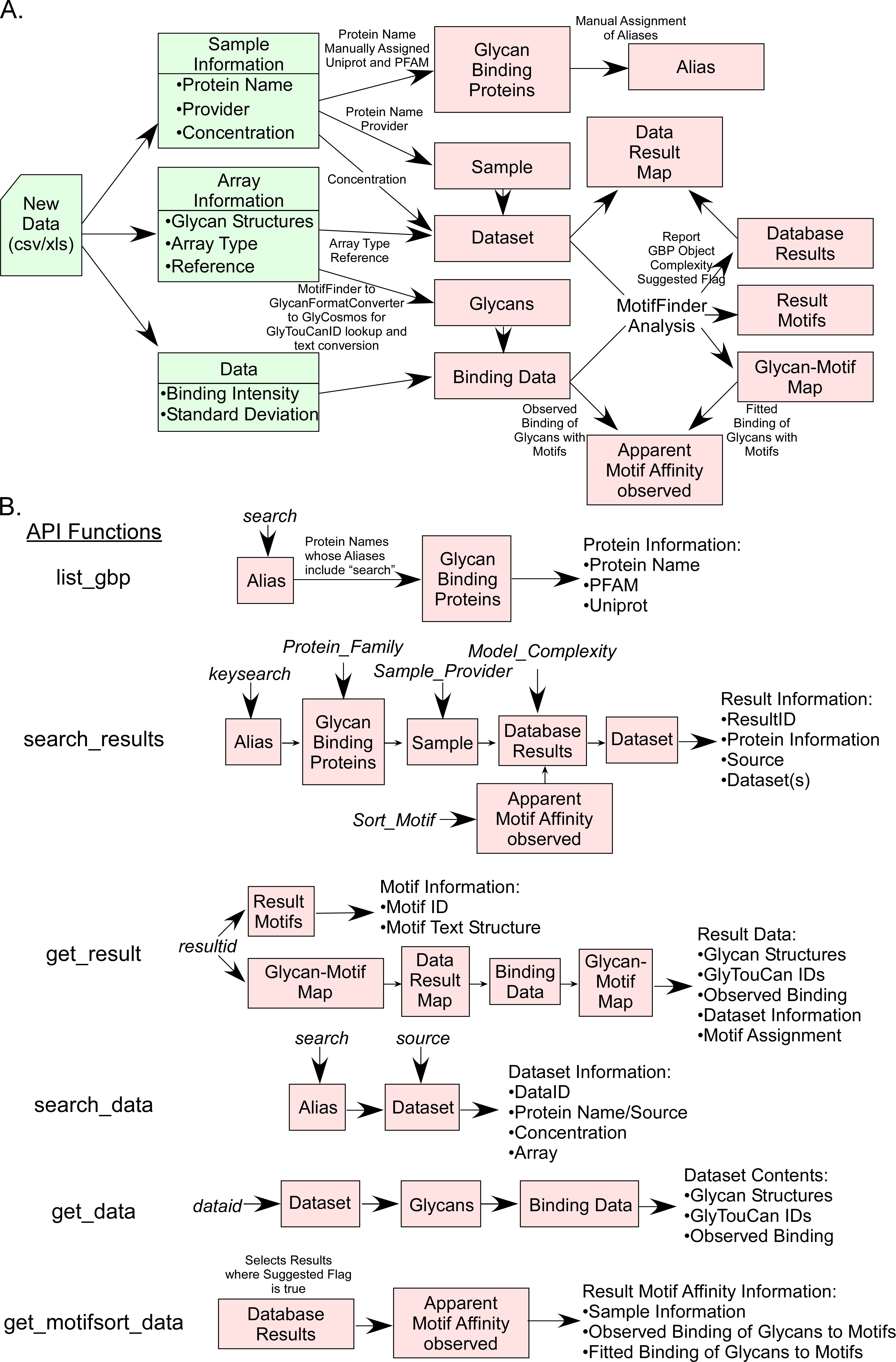

Supplement: SupplementalFigure_S2_cwac022 [file supplementalfigure_s2_cwac022.jpeg]
